# Cartilage Destruction by Hemophilic Arthropathy Can Be Prevented by Inhibition of the Ferroptosis Pathway in Human Chondrocytes

**DOI:** 10.3390/jcm13020559

**Published:** 2024-01-18

**Authors:** Nele Wagener, Sebastian Hardt, Matthias Pumberger, Friederike Schömig

**Affiliations:** Center for Musculoskeletal Surgery, Charité-University Medicine Berlin, Charitéplatz 1, 10117 Berlin, Germany

**Keywords:** hemophilic arthropathy, ferroptosis, cartilage destruction, bone defects, hemophilia, hemarthrosis

## Abstract

(1) Background: Around 50% of hemophilia patients develop severe arthropathy, with even subclinical hemorrhage in childhood potentially leading to intra-articular iron deposition, synovia proliferation, neoangiogenesis, and eventual damage to articular cartilage and subchondral bone. Treatments typically include coagulation factor substitution, radiosynoviorthesis, and joint replacement for advanced cases. This study aims to elucidate programmed cell death mechanisms in hemophilic arthropathy (HA) to identify novel treatments. (2) Methods: Human chondrocytes were exposed to lysed/non-lysed erythrocytes, ferroptosis inducer ML-162, cytokines (IL-1ß, TNFα), and ferric citrate, then assessed for metabolic activity, DNA content, and cell death using Alamar Blue, cyQUANT, and Sytox assays. Three-dimensional spheroids served as a cartilage model to study the effects of erythrocytes and ML-162. (3) Results: Erythrocytes caused significant cell death in 2D cultures (*p* < 0.001) and damaged 3D chondrocyte spheroids. Iron citrate and erythrocytes reduced chondrocyte DNA content (*p* < 0.001). The ferroptosis pathway was implicated in cell death, with no effects from apoptosis and necroptosis inhibitors. (4) Conclusions: This study offers insights into HA’s cell death pathway, suggesting ferroptosis inhibitors as potential therapies. Further studies are needed to evaluate their efficacy against the chronic effects of HA.

## 1. Introduction

The World Federation of Hemophilia (WFH) reports 256,840 registered hemophilia patients globally [1]. Hemophilia, an inherited clotting disorder due to deficiencies in clotting factors VIII or IX, precipitates spontaneous joint bleeding in children, leading to an inflammatory response in the synovial membrane caused by iron accumulation, termed hemophilic arthropathy (HA) [2]. Pathophysiological factors include hemosiderin accumulation, inflammatory mediators, macrophages, oncogenic growth signals, and the excessive vascularization of the synovium [2,3]. This pathology creates a destructive cycle of recurrent bleeding, cartilage damage, and inflammation. Disrupted bone homeostasis, stemming from an unbalanced RANK/RANKL/osteoprotegerin pathway and pro-inflammatory cytokines, leads to subchondral bone defects [2]. If left unaddressed, this can result in joint stiffness, deformity, and ultimately, irreversible joint damage [4].

Recent studies have highlighted the complexity of HA, emphasizing the role of recurrent hemarthrosis in inducing synovial hyperplasia, and the production of pro-inflammatory cytokines such as TNF-alpha, interleukin-6, and 1-beta. These cytokines amplify fibroblast-like synoviocyte proliferation and the production of reactive oxygen species that induce chondrocyte apoptosis, inevitably leading to osteochondral damage due to the direct exposure of chondrocytes to iron, metalloproteinases, and a disintegrin and metalloproteinase with thrombospondin motifs (ADAMTS) produced by fibroblast-like synoviocytes when stimulated by inflammation [3,5].

In addition, advancements in hemophilia treatment over the past decade have significantly improved medium and long-term patient outcomes. The universal use of safer, more effective, and prolonged prophylactic treatments has shown potential in preventing bleeding, and consequently, the development of hemarthrosis and joint damage [6,7,8,9]. The pathogenesis of HA involves a vicious cycle of synovial inflammation, cartilage degeneration, and bone damage, with hemarthrosis promoting synovial hypertrophy and neoangiogenesis, increasing the susceptibility to further bleeding and mechanical damage. The inflamed synovium and direct blood exposure affect the cartilage through cytokine and metalloproteinase release and hydroxyl radical formation, leading to chondrocyte apoptosis [6,7,9,10].

Considering its pathogenesis, potential targets for disease-modifying therapy in HA include iron, inflammation, vascular remodeling, hyperfibrinolysis, bone remodeling, and cartilage regeneration [11]. Although promising in preclinical settings, translating these interventions into clinical practice remains a significant challenge. Key hurdles include establishing a universal outcome measure to predict human efficacy and determining the optimal timing and administration route for these therapies.

Current treatments for hemophilia A include clotting factor replacement, anti-inflammatory medications, radiosynoviorthesis [12], as well as prophylaxis with monoclonal FVIII-mimetic antibody in patients with and without inhibitors [13,14], and chemical synoviorthesis [15,16]. In severe cases where conventional treatments fail to provide adequate relief, surgical options like joint replacement or arthrodesis may be necessary. However, it is important to clarify that infection eradication is specifically critical prior to revising infected prostheses, rather than during initial joint replacement surgeries.

This targeted approach ensures that any pre-existing infections are comprehensively treated to enhance the success and longevity of the surgical correction [17]. However, the specific mechanisms behind blood-mediated cartilage damage in HA remain not fully understood. Apoptosis driven by increased pro-inflammatory cytokines, such as IL-1ß and TNFα, is a recognized pathway leading to chondrocyte death [2]. Direct blood damage through hemosiderin accumulation also plays a significant role in this process [2]. Moreover, recent studies suggest that other forms of regulated cell death, including ferroptosis, might also contribute to the damage of cartilage and the surrounding synovitis. Ferroptosis, distinct from apoptosis, is an iron-dependent form of regulated cell death, characterized by the accumulation of lipid hydroperoxides to lethal levels [18]. This pathway has been increasingly recognized in various pathological conditions, including HA, where excess iron due to recurrent bleeding might trigger ferroptosis in chondrocytes. The exploration of ferroptosis and its potential inhibitors, such as ferrostatin-1 (Fer-1), deferoxamine (DFO), and alpha-tocopherol (aTOH), provides new avenues for therapeutic intervention in HA [19].

Furthermore, advancements in the understanding of HA’s pathogenesis have highlighted the importance of targeting both inflammation and iron overload. Inflammatory pathways, particularly those involving TNFα and IL-1ß, play a crucial role in synovial inflammation and joint destruction [20]. Simultaneously, the management of iron overload through chelation therapy or other means could mitigate the direct cytotoxic effects of hemosiderin on joint tissues [20]. These insights pave the way for more effective targeted therapies that could bridge the gap between current symptomatic treatments and a more holistic approach to managing HA [11].

Ferroptosis, a recently recognized form of regulated cell death (RCD) that is iron-dependent and distinct from apoptosis [21], involves the excessive accumulation of lipid hydroperoxides formed from free hydroxyl radicals and polyunsaturated fatty acids [21,22]. This type of cell death has gained attention due to its unique biochemistry and potential role in various diseases, including neurodegenerative disorders and cancers [22]. Despite its importance, the literature addressing ferroptosis as a potential mechanism for joint tissue damage, particularly in HA, is scarce [22]. HA, often resulting from excess intra-articular iron release into human chondrocytes, may involve cell death mechanisms like ferroptosis, which have not been fully explored yet [22]. Our study aims to delve into the ferroptosis pathway in the context of joint tissue damage in HA. We focus on investigating potential interventions against cell death induced by erythrocytes in human chondrocytes. Interventions such as ferrostatin-1 (Fer-1), deferoxamine (DFO), and alpha-tocopherol (aTOH) are known to inhibit lipid hydroperoxide formation. These agents could offer a new therapeutic approach to mitigate the harmful effects of iron overload in joint tissues, a pivotal component of HA’s pathology.

Understanding ferroptosis in the context of HA could provide a novel perspective for managing this condition. By identifying effective strategies to inhibit ferroptosis, new therapeutic avenues may be developed to protect articular cartilage from the damaging effects of bleeding and iron accumulation in joints. Such advancements could significantly enhance the quality of life for patients with this debilitating condition.

## 2. Materials and Methods

In our study, the experimental design began with four preliminary experiments, each chosen for its specific relevance to our research goals. The SYTOX Green assay was utilized for its ability to accurately assess cell viability and cytotoxicity, crucial for understanding the effects of HA on chondrocytes. The erythrocyte nutrient solution evaluation was conducted to determine its impact on the chondrocyte metabolism and viability, a key factor in joint health. Additionally, the metabolic activity assay with Alamar Blue and the CyQUANT DNA quantification assay were employed to provide a comprehensive understanding of the cellular metabolism and proliferation rates. Following these, this study progressed to two main experiments: investigating the antagonists for ferroptosis, apoptosis, and necroptosis, to explore cellular death pathways in HA, and the fluorescence staining of cryosectioned spheroids, to visually analyze the cellular structures and responses. These experiments were integral in uncovering critical cellular mechanisms and responses in the context of HA.

### 2.1. Cell Culture

Samples were collected from non-hemophilic patients undergoing minimally invasive knee surgery, with ethical approval (EA2/089/20). Chondrocytes (Table 1) were grown in low-glucose DMEM (Gibco, Waltham, MA, USA) with supplements, at 37 °C in a 5% CO_2_ atmosphere.

### 2.2. Substances

This study used ML-162 (25 mg/mL), zVAD-FMK (20 mM), Nec-1 (0.5 mg/L), Fer-1 (10 mg/mL), DFO (5 mg/mL), and aTOH in DMSO (Cayman Chemicals, Ann Arbor, MI, USA; Santa Cruz Biotechnology, Dallas, TX, USA; AdipoGen Life Sciences; San Diego, CA, USA; Sigma-Aldrich Chemie GmbH; Taufkirchen, Germany; WAK-Chemie Medical GmbH; Steinbach, Germany). IL-1ß (10 ng/mL), TNFα (10 ng/mL), and ferric citrate (0.01 g/mL) (Sigma-Aldrich Chemie GmbH; Taufkirchen, Germany) were also applied. SYTOX Green Nucleic Acid stain (5 mM) was utilized to label dead cells, and SYTOX Deep Red Nucleic Acid stain (1 mM) was employed for counterstaining the spheroids. A 10× Triton-X100 (0.05–0.15%) permeabilization buffer was applied to ascertain complete cell death (Thermo Fisher Scientific, Waltham, MA, USA; Sigma-Aldrich Chemie GmbH; Taufkirchen, Germany).

#### 2.2.1. Dilution and Lysis of Erythrocyte Concentrates

Washed human red cell concentrates were filtered for purity (>99%), centrifuged to remove nutrient solution, and lysed. The sediment was diluted to concentrations of 50%, 25%, and 12.5% *v*/*v* in culture medium (Institute for Transfusion Medicine of the Charité-Universitätsmedizin Berlin, Berlin, Germany).

#### 2.2.2. Chondrogenic 3D Spheroid Culture

Spheroids, derived from human chondrocytes (500,000 cells/well) (Table 1), were centrifuged (300× *g*) and cultured for 21 days in a chondrogenic differentiation medium (CDM), with medium changes every three days in 96-well plates (Axygen, Sigma-Aldrich GmbH; Taufkirchen, Germany). Negative controls were maintained in CDM without TGF-beta1.

### 2.3. Fluorometric Cytotoxicity and Proliferation Detection Using SYTOX Green Assay

SYTOX Green, binding DNA upon cell membrane rupture, indicated cell death with enhanced fluorescence (Tecan, InfinitePro2000, Mannedorf, Switzerland). Human chondrocytes (5000/well) in 96-well plates were treated with lysed and non-lysed erythrocytes (50% *v*/*v*), ferric citrate (150 µM) [26], IL-1ß (15 ng/mL) [27,28], TNFα (15 ng/mL) [28], and ML-162 (5 µM) [29]. Fluorescence was measured at 24, 48, and 72 h post-treatment, with cells first stained with SYTOX Green (2 µM) and then permeabilized with Triton-X100 (0.1% *v*/*v*). Preliminary tests confirmed that the selected concentrations and permeabilization times were optimal.

#### 2.3.1. Metabolic Activity Assay with Alamar Blue

The Alamar Blue assay (Thermo Fisher Scientific; Waltham, MA, USA) assessed cell metabolism by detecting fluorescence from viable cells [30]. Chondrocytes (5000/well) were treated with varying concentrations of lysed and non-lysed erythrocytes, IL-1ß, TNFα, ferric citrate, and ML-162 in 48-well plates. Fluorescence (ex:560 nm/em:590 nm) was measured at 24, 48, and 72 h post-treatment using a Tecan plate reader (Tecan, InfinitePro2000) to quantify metabolic activity.

#### 2.3.2. CyQUANT DNA Quantification Assay

The CyQuant assay was employed to validate the Alamar Blue assay’s metabolic activity findings. This sensitive fluorescence-based method measures DNA content to accurately quantify cell proliferation and cytotoxicity. Chondrocytes (5000/well) in 48-well plates were treated with erythrocytes, IL-1ß, TNFα, ferric citrate, and ML-162, mirroring the Alamar Blue conditions. Post 24, 48, and 72 h incubation, cells were lysed and stained with CyQuant, with fluorescence (ex:504 nm/em:523 nm) measured to determine cell quantity.

#### 2.3.3. Antagonists for Ferroptosis, Apoptosis, and Necroptosis

Human chondrocytes in 96-well plates were treated with ferroptosis inducer ML-162 (5 µM) [29], inhibitors Fer-1 (1 µM) [31,32,33], DFO (20 µM) [30,34], aTOH (20 µM) [35,36,37], apoptosis inhibitor zVAD-fmk (20 µM) [31,38], and necroptosis inhibitor Nec-1 (20 µM) [39] 30 min before erythrocyte exposure. Post-treatment, cells were stained with Sytox green (2 µM) for fluorescence quantification via a Tecan plate reader.

#### 2.3.4. Fluorescence Staining of Cryosectioned Spheroids

Chondrogenic spheroids (500,000 cells/well) were treated with erythrocytes and ML-162 (5 µM) for 72 h, stained with SYTOX green (2 µM), and fixed in PFA (4%). They were then embedded in gelatin, marked with PROLENE (Johnson & Johnson GmbH; Neuss, Germany), and frozen in Tissue Tek (Plano GmbH; Wetzlar, Germany). Post-sectioning, spheroids were counterstained with SYTOX Deep Red (1 mM) for nucleus visualization.

#### 2.3.5. Hematoxylin and Eosin Staining

Cryosections (5–10 µm) were prepared at −25 °C, attached to slides (New Erie Scientific LLC; Portsmouth, NH, USA), and fixed with 4% formaldehyde. Subsequently, they were stained using Harris’s hematoxylin and eosin (Sigma-Aldrich Chemie GmbH; Taufkirchen, Germany) for morphological analysis, and nuclei distribution was observed using light microscopy (Leica DMi8, Wetzlar, Germany).

### 2.4. Morphological Assessment of Erythrocyte-Induced Damage Depth of the Spheroids

Microscopy was conducted using a Leica SP5 confocal microscope, and images were captured with an Olympus DP27 camera and Olympus cellSens software (version 4.2, Olympus, Tokyo, Japan). The extent of damage to the spheroids was comparatively analyzed.

#### Software and Statistical Analysis

Metric measurements are summarized as medians (Med) and interquartile ranges (IQR). The Shapiro–Wilk test was used to detect the nonnormality of scales. Skewness is a measure of symmetry, or more precisely, the lack of symmetry of the normal distribution. Kurtosis is a measure of the peakedness of a distribution. Skewness and kurtosis are used to assess the shape of the distribution. If the absolute values are less than 1, the deviation from the normal distribution is considered robust with respect to parametric test procedures [40]. A total of 36 samples of 6 different chondrocyte cell donors and 6 technical replicates are available as a basis for each experimental setting. Measurements are considered as dependent within the donors, and as independent between the donors. The Friedman Test was applied to test the difference of paired samples. In cases of significance, Wilcoxon paired samples post-hoc tests were performed. The Bonferroni adjustment was applied for multiple testing. Boxplots were used in graphical representations, based on the five-number summary—minimum value, 25th percentile, median (50th percentile), 75th percentile, and maximum value. Outliers may be indicated beyond the extreme values by dots or asterisks exceeding 1.5 or 3 times the box length. Analyses were performed using SPSS version 29 (IBM Corp., Armonk, NY, USA), and *p*-values < 0.05 were considered statistically significant.

## 3. Results

### 3.1. Erythrocyte Nutrient Solution Maintains Stability in the Chondrocyte Environment

**Figure 1:**
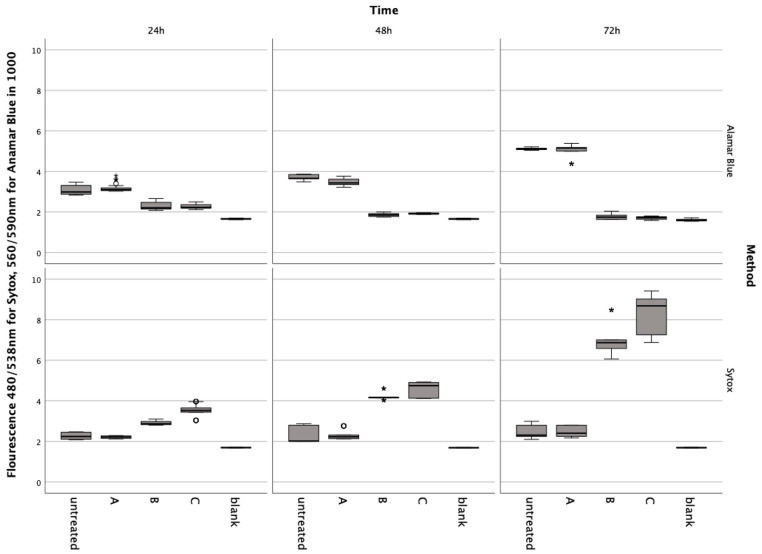
Results from the Alamar Blue and Sytox Green assays conducted on chondrocytes incubated with erythrocyte concentrate for 24, 48, and 72 h. Data are shown as boxplots. In a boxplot, circles indicate mild outliers, data points exceeding 1.5 times the interquartile range (IQR) from the quartiles, while asterisks mark extreme outliers, which are significantly further from the median, beyond 3 times the IQR. A: ERY nutrient medium, B: ERY non-lysed, C: ERY lysed.

In preliminary tests, it was shown that the nutrient solution contained in the red cell concentrates did not induce metabolic changes or cell death in the chondrocytes (Figure 1). The Friedmann test for Alamar Blue and Sytox showed significant differences at each incubation time ($\chi^{2}$(4) < 0.001). The Post-hoc Wiscoxon test showed no significant difference between the untreated group and A: ERY nutrient medium for Alamar Blue at 24 h (Z = −0.911, *p* = 0.369), 48 h (Z_adj_ = −0.523, p_adj_ = 0.699), and 72 h (Z = −0.086, *p* = 0.935) and Sytox at 24 h (Z_adj_ = −0.288, p_adj_ = 0.613), 48 h (Z = −0.613, *p* = 0.547) and 72 h (Z = −0.283, *p* = 0.783).

### 3.2. H&E-Stained Distribution of the Cell Nuclei

**Figure 2:**
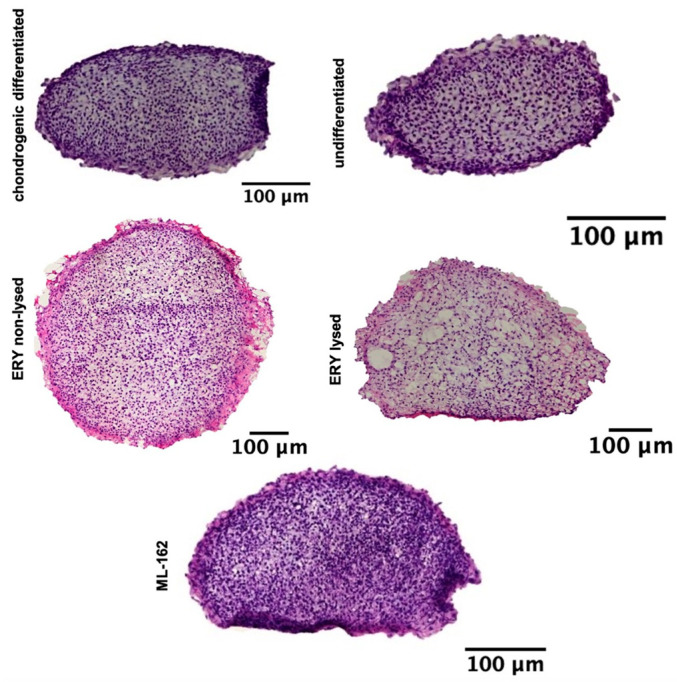
Illustrates a 21-day chondrogenesis of spheroids, with H&E stains of treated and untreated groups. Scale bar: 100 µM.

H&E staining of cell nuclei distribution revealed a consistent and dense cell distribution across the entire surface of the spheroid sections for the chondrogenic differentiated, nondifferentiated, non-lysed erythrocytes, lysed erythrocytes, and ML-162 spheroids (Figure 2).

### 3.3. Influence of Lysed and Non-Lysed Erythrocytes on Chondrocyte Metabolic Dynamics

**Figure 3:**
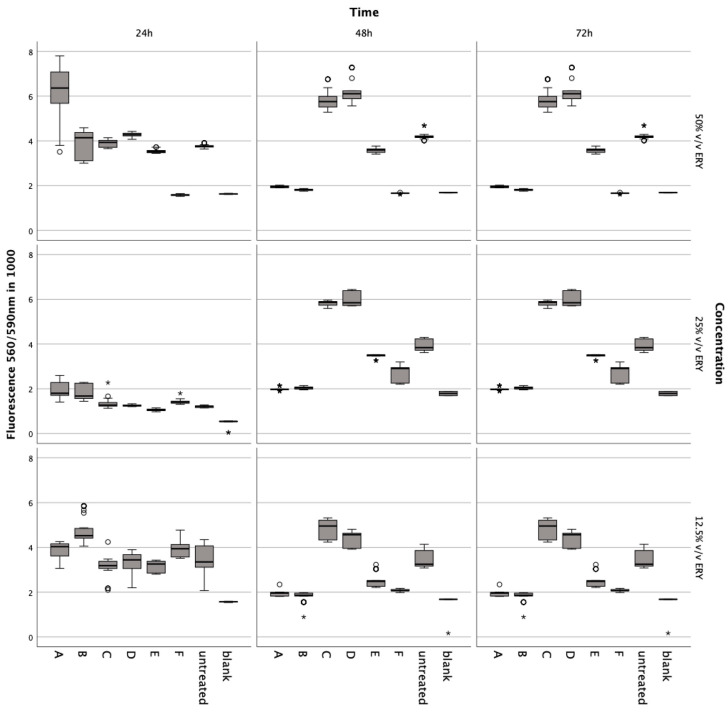
Metabolic impact on human chondrocytes over 72 h, assessed by the Alamar Blue assay after exposure to varying concentrations of intact and lysed erythrocytes (ERY; 12.5%, 25%, 50% *v*/*v*), IL-1. (5, 10, 15 ng/mL), TNFα (5, 10, 15 ng/mL), ML-162 (5 μM), and Ferric citrate (100 μM). Data are shown as boxplots. In a boxplot, circles indicate mild outliers, data points exceeding 1.5 times the interquartile range (IQR) from the quartiles, while asterisks mark extreme outliers, which are significantly further from the median, beyond 3 times the IQR. A: 50% *v*/*v* ERY lysed, B: 50% *v*/*v* ERY non-lysed, C: IL-1ß, D: TNFα, E: 12.5% *v*/*v* ERY lysed; Ferric citrate (100 µM); IL-1ß (10 ng/mL), F: ML-162 (5 µM).

Incubation with lysed and non-lysed erythrocytes at 50% *v*/*v* (Z = −5.233, *p* < 0.001; Z = −5.232; *p* < 0.001), 25% *v*/*v* (Z = −5.233, *p* < 0.001; Z = −5.232, *p* < 0.001), and 12.5% *v*/*v* (Z = −5.232, *p* < 0.001) for 72 h significantly reduced chondrocyte metabolic activity (Figure 3).

A noticeable decline was observed at 50% *v*/*v* (Z = −5.233, *p* < 0.001; Z = −5.232, *p* < 0.001) after 48 h compared to controls. The ferroptosis inducer ML-162 at 5 μM also markedly decreased metabolism after 24 h (Z = −5.232, *p* < 0.001), 48 h (Z = −5.232, *p* < 0.001), and 72 h (Z = −5.232, *p* < 0.001). Conversely, IL-1ß and TNFα elevated metabolic activity after 72 h at 5 ng/mL (Z = −5.232, *p* < 0.001), 10 ng/mL (Z = −5.232, *p* < 0.001; Z = −5.233, *p* < 0.001), and 15 ng/mL (Z = −5.232, *p* < 0.001; Z = −4.997, *p* < 0.001) compared to untreated chondrocytes. Ferric citrate, simulating iron overload, significantly reduced metabolism at 50 μM (Z = −5.234, *p* < 0.001) and 150 μM (Z = −5.122, *p* < 0.001) over 72 h relative to untreated cells.

### 3.4. Exploring the Impact of Erythrocyte Conditions on Chondrocyte Survival

**Figure 4:**
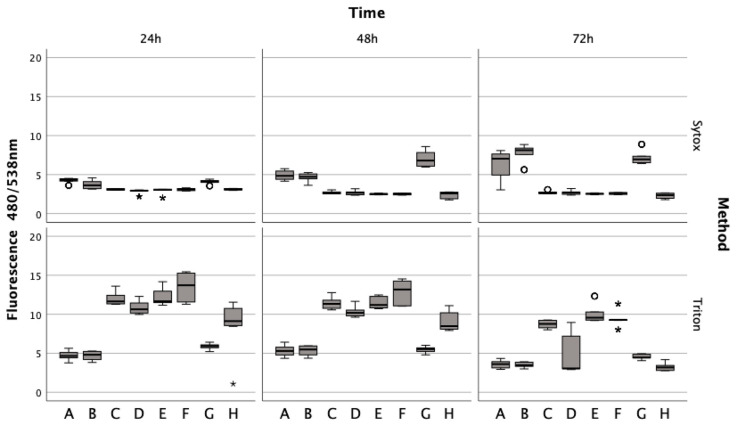
Effects of lysed/non-lysed erythrocytes (ERY), cytokines IL-1. and TNFα, ML-162, and Ferric citrate on human chondrocyte cell death over 72 h, as measured by Sytox green. TritonX-100 confirmed complete cell death. In a boxplot, circles indicate mild outliers, data points exceeding 1.5 times the interquartile range (IQR) from the quartiles, while asterisks mark extreme outliers, which are significantly further from the median, beyond 3 times the IQR. A: 50% *v*/*v* ERY lysed, B: 50% *v*/*v* ERY non-lysed, C: untreated, D: Ferric citrate (150 µM), E: IL-1ß (15 ng/mL), F: TNFα (15 ng/mL), G: ML-162 (5 µM), H: 50% *v*/*v* ERY non-lysed; Ferric citrate (150 µM); IL-1ß (15 ng/mL).

Lysed and non-lysed erythrocytes induce chondrocyte cell death. Sytox green staining revealed that chondrocytes treated with lysed and non-lysed erythrocytes for 48 h (Z = −5.232, *p* < 0.001; Z = −5.233, *p* < 0.001) and 72 h (Z = −5.233, *p* < 0.001) exhibited significantly increased cell death compared to untreated chondrocytes (Figure 4). ML162 at 5 μM significantly prompted cell death at all observed time points (Z = −5.234, *p* < 0.001; Z = −5.233, *p* < 0.001), more so than erythrocyte treatments. However, IL-1ß and TNFα at 15 ng/mL did not significantly affect cell death at 24 h (Z_adj_ = −0.081, p_adj_ = 0.532; Z = −0.283, *p* = 0.783), 48 h (Z = −0.613, *p* = 0.547; Z_adj_ = −0.143, p_adj_ = 0.557), and 72 h (Z = −0.047, *p* = 0.966; Z_adj_ = −0.161, p_adj_ = 0.564). For comparison, Triton X-100 served as a positive control, achieving 100% cell death by permeabilizing living cells.

Untreated chondrocytes (Z = −5.232, *p* < 0.001; Z = −5.233, *p* < 0.001) and those treated with IL-1ß (Z = −5.232, *p* < 0.001; Z = −5.233, *p* < 0.001; Z = −5.234, *p* < 0.001) or TNFα (Z = −5.233, *p* < 0.001; Z = −5.232, *p* < 0.001; Z = −5.234, *p* < 0.001) displayed significant fluorescence after permeabilization with Triton at all time points, unlike cells treated with erythrocytes or ML-162. Additionally, chondrocytes showed a marked decrease in the fluorescence signal after a 72 h ferric citrate exposure (150 μM) post-permeabilization with Triton, similar to the erythrocyte (Z = −0.519, *p* < 0.611) and ML-162 groups (Z = −0.518, *p* < 0.611).

### 3.5. Assessing the Influence of Iron Citrate and Erythrocytes on Chondrocyte DNA Content

**Figure 5:**
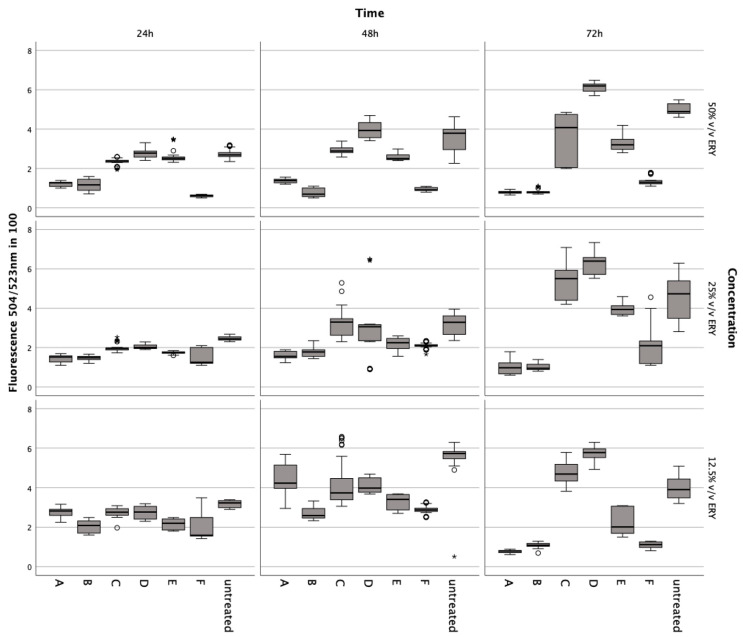
CyQuant assays to evaluate the impact of various concentrations of lysed/non-lysed erythrocytes, IL-1., TNFα, ML-162, and ferric citrate on human chondrocytes’ DNA content, indicating proliferation over 72 h. In a boxplot, circles indicate mild outliers, data points exceeding 1.5 times the interquartile range (IQR) from the quartiles, while asterisks mark extreme outliers, which are significantly further from the median, beyond 3 times the IQR. A: 50% *v*/*v* ERY lysed, B: 50% *v*/*v* ERY non-lysed, C: IL-1ß (15 ng/mL), D: TNFα (15 ng/mL), E: Ferric citrate (150 µM), F: ML-162 (5 µM).

Both lysed and non-lysed erythrocytes decreased DNA significantly at 50% *v*/*v* after 24 h (Z = −5.232, *p* < 0.001; Z = −5.233, *p* < 0.001), 48 h (Z = −5.232, *p* < 0.001), and 72 h (Z = −5.234, *p* < 0.001; Z = −5.233, *p* < 0.001), at 25% *v*/*v* after 24 h (Z = −5.233, *p* < 0.001), 48 h (Z = −5.233, *p* < 0.001; Z = −5.232, *p* < 0.001), and 72 h (Z = −5.232, *p* < 0.001; Z = −5.233, *p* < 0.001), at 12.5% *v*/*v* after 24 h (Z = −4.620, *p* < 0.001; Z = −5.233, *p* < 0.001), 48 h (Z = −4.666, *p* < 0.001; Z = −4.981, *p* < 0.001), and 72 h (Z = −5.232, *p* < 0.001) (Figure 5). Ferric citrate at 150 μM also significantly lowered DNA at all points of measurement (Z = −5.233, *p* < 0.001; Z = −5.083, *p* < 0.001; Z = −5.147, *p* < 0.001; Z = −4.668, *p* < 0.001; Z = −3.496, *p* < 0.001; Z = −5.232, *p* < 0.001).

Conversely, incubation of chondrocytes with TNFα and IL-1ß at 15 ng/mL increased DNA content after 72 h (Z = −5.122, *p* < 0.001; Z = −5.232, *p* < 0.001; Z = −3.096, *p* = 0.001; Z = −4.914, *p* < 0.001; Z = −3.433, *p* < 0.001).

### 3.6. Ferroptosis Inhibitors Show Promising Protection for Chondrocytes against Erythrocyte Induced Stress, beyond Apoptosis and Necroptosis Pathways

**Figure 6:**
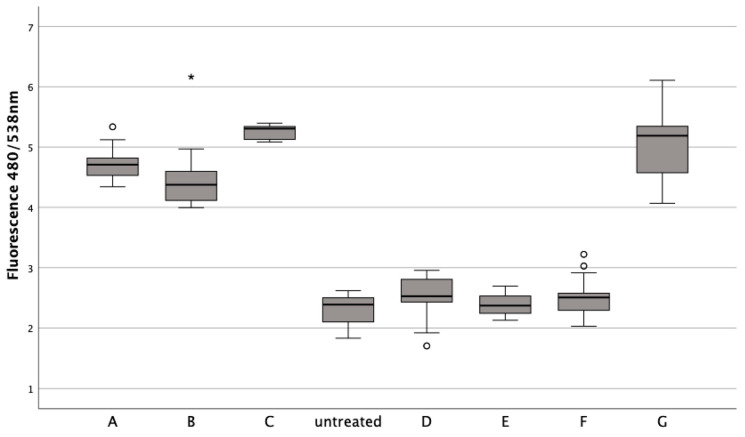
The impact of ferroptosis inhibitors (Fer-1, DFO, aTOH), lysed erythrocytes (50% *v*/*v*), and ML-162 (5 μM) on the death of human chondrocytes over a 24 h period. In a boxplot, circles indicate mild outliers, data points exceeding 1.5 times the interquartile range (IQR) from the quartiles, while asterisks mark extreme outliers, which are significantly further from the median, beyond 3 times the IQR. ML-162 (5 µM), B: zVAD (20 µM) + ERY, C: Nec-1 (20 µM) + ERY, D: Fer-1 (1 µM) + ERY, E: DFO (20 µM) + ERY, F:aTOH (20 µM) + ERY, G: 50% *v*/*v* ERY lysed.

Chondrocytes treated with erythrocytes showed a significant reduction in cell death when ferroptosis inhibitors Fer1 (1 μM) (Z = −5.232, *p* < 0.001), DFO (20 μM) (Z = −5.232, *p* < 0.001), or aTOH (20 μM) (Z = −5.232, *p* < 0.001) were added after 24 h of incubation, similar to untreated chondrocytes (Z = −2.545, *p* = 0.010; Z = −1.312, *p* = 0.193; Z = −1.854, *p* = 0.064) (Figure 6). ML-162, on the other hand, significantly increased the fluorescence signal compared to the untreated control (Z = −5.232, *p* < 0.001). Apoptosis inhibitor zVAD (20 μM) (Z_adj_ = −0.143, p_adj_ = 0.557) and necroptosis inhibitor Nec-1 (20 μM) (Z = −1.571, *p* = 0.118) resulted in a fluorescence signal similar to that of the lysed erythrocytes when used at a concentration of 50% *v*/*v* after a 24 h incubation with lysed erythrocytes.

### 3.7. Observing Erythrocyte Influence on Chondrogenic Spheroid Integrity

**Figure 7:**
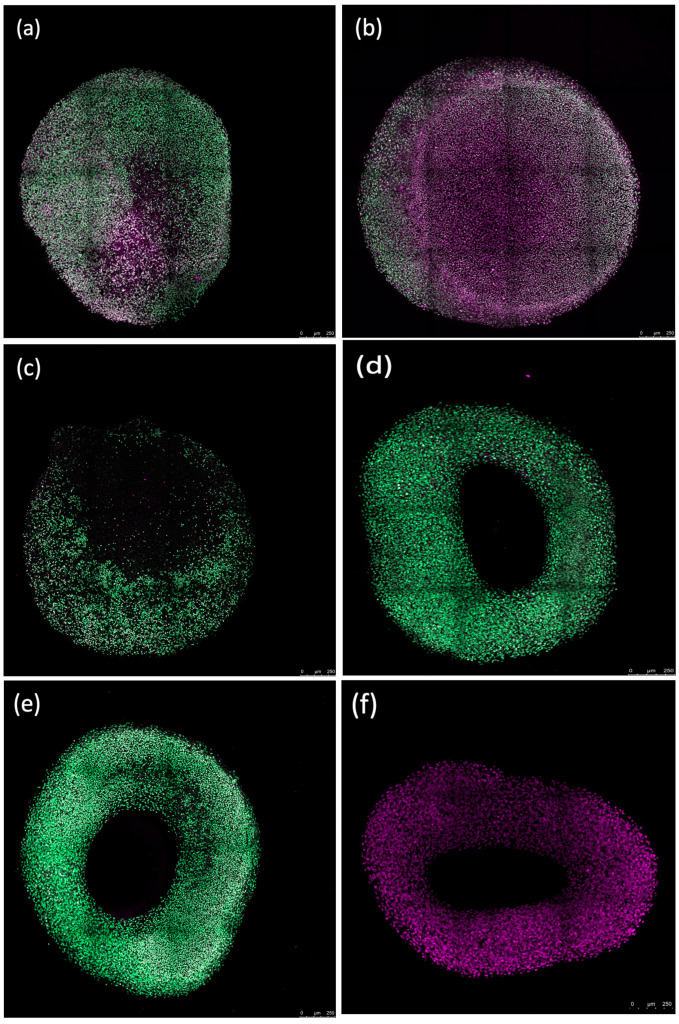

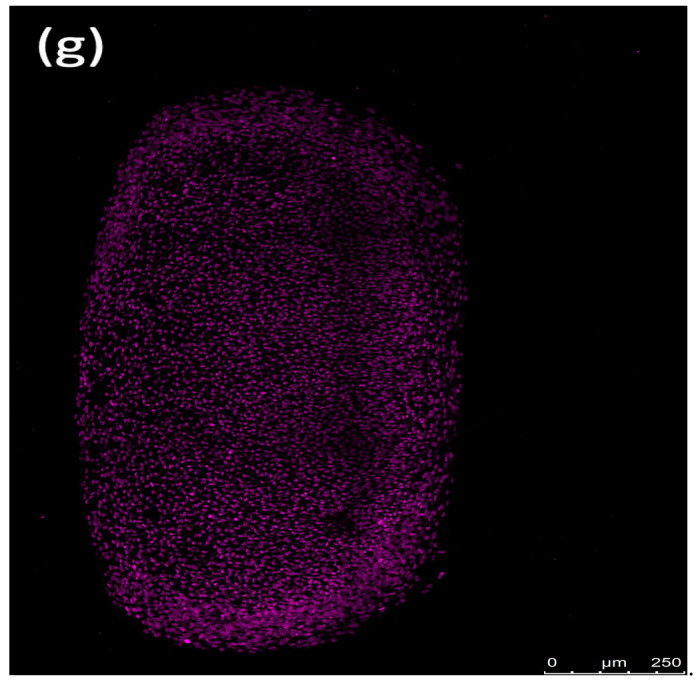
Confocal images showing the impact of 50% lysed (**a**,**d**) and non-lysed (**b**,**e**) red blood cells and 5 µM ML-162 (**c**) on human chondrogenic spheroids over 72 h. Untreated chondrogenic (**f**) and non-chondrogenic (**g**) spheroids are also shown. Scale bar: 250 µM.

Fluorescence imaging revealed that erythrocytes caused peripheral damage in spheroids, with a notable decrease in Sytox green fluorescence at the core of the spheroid after a 72 h incubation. Counterstaining with Sytox deep red highlighted the red-stained regions at the spheroid’s center (Figure 7). The periphery of the spheroids treated with the ferroptosis inducer ML-162 exhibited extensive cell death, as evidenced by robust Sytox green staining, whereas the center showed reduced staining intensity. In contrast, both the differentiated and non-differentiated spheroids without erythrocyte exposure displayed no Sytox green staining, instead showing uniform Sytox deep red staining of fixed nuclei across the spheroid’s surface.

## 4. Discussion

This in vitro research initiates an exploration into the effects of erythrocytes on human chondrocytes, illuminating the cellular death processes associated with HA. By using washed human red cell concentrates in escalating concentrations, we replicated the iron overload that characterizes HA, typically marked by repeated joint hemorrhages [2].

Currently, joint hemorrhage is recognized as the catalyst for HA, with elements like hemosiderin, inflammatory cytokines, macrophages, oncogenes, and excessive vascular proliferation within the joint mucosa contributing to its development. In late-stage HA, extensive cartilage loss and subchondral bone sclerosis severely impair joint function, leading to restricted movement, crepitus, and deformities in patients [2]. Normally, macrophage-like synoviocytes absorb hemosiderin and ferritin after trauma-induced bleeding and recycle them into the bloodstream. However, in HA, recurrent hemarthrosis causes synovial macrophages to accumulate blood breakdown products, eventually surpassing the synovial membrane’s capacity to expel these substances, resulting in iron buildup and synovial hypertrophy [2].

Our investigation into erythrocyte-induced cell death encompassed three distinct methodologies and identified the implicated signaling pathways through the use of different antagonists. The main result of this study on cell death pathways, specifically ferroptosis, in the context of HA is that ferroptosis inhibitors, notably Fer-1, DFO, and aTOH, effectively prevent erythrocyte-induced cell death in chondrocytes. This effect is independent of the apoptosis and necroptosis pathways.

Our primary findings reveal a pronounced induction of chondrocyte cell death in two dimensions, instigated by a 24 h exposure to erythrocytes. To demonstrate erythrocyte-induced damage, we developed three-dimensional chondrocyte spheroids containing 500,000 cells. The severity of the damage was apparent within 72 h. These findings underscore the significant impact of ferroptosis in the progression of HA induced by the accumulation of erythrocytes. This suggests that erythrocytes may inflict damage on both articular cartilage and subchondral bone. In cases of HA where bleeding persists, synovial fluid reaches its capacity to cleanse, resulting in iron accumulation and the synovial membrane’s hyperplasia [2]. Our findings indicate that ferric citrate treatments resulted in significant iron-induced chondrocyte cell death, mirroring the effects seen with erythrocyte treatments. This supports the notion that iron overload contributes to cartilage damage in HA.

Notably, in our study, the inflammatory mediators IL-1ß and TNFα significantly boosted cell metabolism and DNA synthesis after 72 h, which contrasts with previous research where these cytokines have been implicated in cartilage degradation due to their role in inducing chondrocyte death and releasing destructive enzymes and nitric oxide from immune cells [41]. The unexpected chondroprotective effects of TNFα and IL-1ß observed in our study may be attributed to the lack of synovial and immune cells in the experimental setup. This aligns with the findings of Relic et al., who demonstrated that TNFα pre-treatment could shield chondrocytes from cell death induced by the proapoptotic agent sodium nitroprusside, suggesting a context-dependent role of these cytokines in chondrocyte viability [41].

In 2021, Yao et al. discovered that ferroptosis in chondrocytes could be triggered by ferric ammonium citrate (FAC) and IL-1ß in mice, with the antioxidant Fer-1 slowing cartilage degradation in osteoarthritis [26]. Our findings diverge; in human chondrocytes, we successfully inhibited erythrocyte-induced ferroptosis using antagonists Fer-1, DFO, and aTOH, potentially curbing cartilage damage progression in HA. Contrary to Yao et al.’s results, where IL-1ß induced ferroptosis, our study observed that IL-1ß actually stimulated cell growth. The role of IL-1ß merits further discussion; it appears to have variable effects depending on the cell type, cytokine concentration, and duration of exposure. While some research indicates IL-1ß may encourage chondrocyte proliferation [42], others suggest it can signal programmed cell death [43]. The complexities of IL-1ß’s impact on chondrocytes suggest it is modulated by various factors, including the local environment, cytokine levels, tissue health, and the presence of other inflammatory or healing agents [42,43].

In our endeavor to discern the types of cell death—ferroptosis, apoptosis, and necroptosis—in HA, we employed a comprehensive approach. The administration of ferroptosis antagonists Fer-1, DFO, and aTOH, alongside the use of zVAD and Nec-1, inhibitors of apoptosis and necroptosis, respectively, provided insightful observations. The failure of zVAD and Nec-1 to block erythrocyte-induced cell death strongly suggests that the erythrocyte-triggered cell death in our study is not attributable to apoptosis or necroptosis. This finding is pivotal as it underscores the unique role of erythrocytes in inducing a specific form of cell death, distinct from the traditionally recognized pathways of apoptosis and necroptosis.

Furthermore, our introduction of ML-162, a GPX4 inhibitor, to induce ferroptosis in chondrocytes led to revealing results. The observation that both ML-162 and erythrocytes trigger chondrocyte cell death with similar levels of fluorescence intensity points to a potent induction of ferroptosis. This is a significant finding, considering the role of GPX4 as a central regulator in ferroptosis, where its inhibition leads to the accumulation of lipid hydroperoxides, a hallmark of ferroptosis.

Our subsequent treatment with the trio of ferroptosis antagonists—Fer-1, DFO, and aTOH—significantly counteracted the erythrocyte-induced cell death, achieving a fluorescence signal comparable to the untreated control. This finding not only corroborates the induction of ferroptosis by erythrocytes but also highlights the potential therapeutic efficacy of these antagonists in mitigating ferroptosis. Our findings align with those of Zilka et al., who demonstrated that Fer-1 suppressed ferroptosis in mice via lipid peroxidation inhibition [44]. Additionally, the work of Dixon et al. with rat brain slices further corroborated the effectiveness of Fer-1 as a robust ferroptosis inhibitor [45]. These studies collectively underscore the therapeutic potential of targeting ferroptosis in HA.

The implications of our findings extend beyond the specific context of HA, offering insights into the broader understanding of ferroptosis as a cell death mechanism. They open avenues for the exploration of ferroptosis inhibitors as potential therapeutic agents in conditions characterized by pathological iron accumulation and oxidative stress. This could pave the way for novel treatment strategies in a range of disorders where ferroptosis plays a key role.

In addition to our earlier interventions, we also incorporated alpha-tocopherol (aTOH), an antioxidant known for its ferroptosis inhibitory properties. This approach aligns with the work of Kagan and Kang et al., who have demonstrated the efficacy of aTOH in preventing ferroptosis [46,47]. aTOH functions by hindering the activity of lipoxygenase (LOX), an enzyme that catalyzes the oxidation of polyunsaturated fatty acids (PUFAs) in peripheral blood mononuclear cells (PBMCs), and in C57BL/6J mice genetically modified to lack the Pfa1 and Gpx4 genes implicated in ferroptosis [44,45,46,47,48]. This inhibition of LOX is crucial as it typically promotes lipid peroxidation, a key process in the initiation of ferroptosis. By preventing lipid peroxidation and the subsequent oxidative stress, aTOH offers a protective mechanism against ferroptotic cell death.

Moreover, our utilization of deferoxamine (DFO), an iron chelator, plays a significant role in curtailing intracellular iron buildup, thereby effectively inhibiting the harmful effects of iron overload [45,49,50]. Iron overload is a known contributor to oxidative stress and cellular damage, particularly in conditions like HA where repeated hemorrhages lead to iron deposition in the joint tissues. DFO’s ability to reduce intracellular iron concentrations is instrumental in mitigating the oxidative stress and subsequent cell death pathways, including ferroptosis.

The collective data from our study, therefore, reinforce the hypothesis that blood-induced cartilage damage in HA is intricately linked to ferroptosis. The efficacy of Fer-1, DFO, and aTOH in counteracting the cartilage alterations caused by erythrocytes supports this theory. These findings are pivotal in understanding the underlying mechanisms of joint damage in HA and open new avenues for targeted therapeutic interventions. Specifically, the modulation of ferroptosis pathways offers a novel and promising approach to mitigate joint damage in this patient population, potentially leading to improved clinical outcomes and enhanced quality of life for individuals affected by this debilitating condition.

While our research provides valuable insights into the role of ferroptosis in HA and its potential therapeutic targets, it is important to acknowledge certain limitations. The study’s in vitro nature limits the direct translation of findings to clinical settings, as cell culture conditions may not fully replicate the complex in vivo environment of human joints. Additionally, the use of chondrocytes from non-hemophilic patients may not fully capture the specific cellular responses characteristic of HA. Future studies, ideally incorporating in vivo models and hemophilia patient-derived cells, are necessary to validate our findings and further explore the intricate mechanisms underlying HA.

Our comprehensive approach to identifying the types of cell death involved has highlighted the unique role of erythrocytes in inducing a specific form of cell death, suggesting that traditional pathways like apoptosis and necroptosis may not be the primary drivers in this context.

The efficacy of ferroptosis antagonists such as Fer-1, DFO, and aTOH in counteracting erythrocyte-induced cell death illuminates the potential therapeutic value of targeting this pathway. This finding not only corroborates the role of ferroptosis in HA, but also suggests these antagonists as promising candidates for mitigating joint damage. The alignment of our findings with the literature further solidifies the position of ferroptosis as a significant cell death mechanism in HA and possibly other related conditions.

## 5. Conclusion

Our findings suggest that Fer-1, DFO, and aTOH could potentially mitigate or inhibit ferroptosis in individuals with hemophilia, paving the way for novel treatment approaches aimed at enhancing patient quality-of-life and alleviating pain, immobility, and psychosocial stress. The efficacy of these ferroptosis inhibitors in counteracting the chronic implications of HA warrants additional in vitro studies and in vivo trials to confirm their long-term therapeutic benefits. This opens up new possibilities for improved patient management, aiming to preserve joint function and minimize the need for surgical interventions.

## Figures and Tables

**Table 1:** Characteristics of Human Chondrocytes Isolated from Non-Hemophilic Patients Undergoing Minimally Invasive Knee Surgery.

Feature	Description
Cell Type	Human Chondrocytes
Source	Isolated from the knee joints of non-hemophilic patients during minimally invasive surgery
Morphology	Round to oval in shape, with a prominent nucleus [23].
Size	Variable, typically ranging from 10–30 µm in diameter [23].
Proliferation Rate	Low; chondrocytes divide slowly [24].
Synthetic Activity	Production of Collagen Type II, Aggrecan, and other extracellular matrix components [25].
Markers	Typical chondrocyte markers such as Collagen Type II, Aggrecan, SOX9
Function	Maintenance of cartilage structure and function through synthesis and regulation of the extracellular matrix [24].

## Data Availability

The raw data supporting the conclusions of this article will be made available by the authors on request.
